# Neutrophils aggravate inflammatory lesions in intestinal organoids from necrotizing enterocolitis

**DOI:** 10.3389/fimmu.2025.1582526

**Published:** 2025-06-27

**Authors:** Kim M. Heuer, Michael Boettcher, Laia Pagerols Raluy, Johanna Hagens, Jan P. Kolman, Madeleine J. Bunders, Jasmin Wesche, Jasmin Knopf, Martin Herrmann, Konrad Reinshagen, Deirdre Vincent

**Affiliations:** ^1^ Department of Pediatric Surgery, University Medical Center Hamburg-Eppendorf, Hamburg, Germany; ^2^ Department of Pediatric Surgery, University Medical Center Mannheim, Heidelberg University, Mannheim, Germany; ^3^ Department of Virus Immunology, Leibniz Institute of Virology, Hamburg, Germany; ^4^ III. Department of Medicine, University Medical Center Hamburg-Eppendorf, Hamburg, Germany; ^5^ Hamburg Center of Translational Immunology, University Medical Center Hamburg-Eppendorf, Hamburg, Germany; ^6^ Department of Medicine 3, Friedrich Alexander University Erlangen-Nuremberg and Universitätsklinikum Erlangen, Erlangen, Germany; ^7^ Deutsches Zentrum Immuntherapie (DZI), Universitätsklinikum Erlangen, Erlangen, Germany

**Keywords:** NEC, neutrophils, intestinal organoids, neonate, TLR-4, innate immune system, co-culture

## Abstract

**Introduction:**

Necrotizing enterocolitis (NEC) is a leading cause of neonatal death and long-term morbidity, involving complex pathophysiology including prematurity, abnormal bacterial colonization, and ischemia-reperfusion injury, partially mediated by neutrophils. However, the limitations of current animal models hinder the development of targeted therapies for NEC. Thus, this study aimed to develop a human intestinal organoid model for NEC to investigate its pathophysiology, understand neutrophil involvement, and bridge animal and human research.

**Methods:**

Organoid cultures were established from human neonatal intestinal samples with NEC (n=7) and without gut inflammation (controls, n=7), treated with lipopolysaccharides (LPS), and/or cocultured with neutrophils. Flow cytometry quantified neutrophil survival (propidium iodide/Annexin-V), activation (CD11b/CD66b), and TLR-4 expression, as well as organoid TLR-4 expression and apoptosis markers. NEC status and neutrophil recruitment were analyzed using immunofluorescence.

**Results:**

After LPS administration, NEC organoids showed significantly increased TLR-4 expression, intestinal apoptosis markers, and NEC scores compared to controls, with more pronounced differences after neutrophil addition. Neutrophil activation markers were elevated when cocultured with both NEC and control organoids, but TLR-4 expression increased only with NEC organoids.

**Discussion:**

The findings suggest that epithelial cells from NEC patients have a heightened innate TLR-4 expression upon LPS stimulation, potentially contributing to NEC development. LPS stimulation resulted in more pronounced NEC-like lesions in NEC organoids, which were exacerbated by neutrophils. This model demonstrates that neutrophils might contribute to NEC manifestation and maintenance and that NEC organoids can reflect disease aspects, potentially aiding in the development of targeted therapies.

## 1 Introduction

Necrotizing enterocolitis (NEC), a devastating inflammatory bowel disease, affects roughly 0.1-0.3% of all newborns. While term-born neonates account for only a few NEC cases, over 90% of NEC cases are diagnosed in preterm-born neonates (1–3). As NEC is most prevalent in neonates with a birthweight below 1500g (rate 7-14%) (3), it is not surprising that NEC incidence rates have risen, as improvements in neonatal care and advances in treatment options result in increased survival of very prematurely born neonates (1, 4–6). As mortality rates range between 15-30% (4, 7), NEC is one of the leading causes of death of preterm neonates (8, 9), with the highest mortality seen in neonates requiring necrotic bowel resection (4, 10).

NEC is characterized by severe intestinal inflammation, which can progress into intestinal wall necrosis. If the bowel ruptures, individuals often develop peritonitis, systemic sepsis, and eventually organ failure (11–13). Currently, NEC management involves antibiotics treatment and discontinuation of enteral feeding; however, in advanced cases (stage IIIb) surgical removal of necrotic intestine is often necessary (14–16). Unfortunately, surgery is frequently associated with long-term complications, such as feeding difficulties, intestinal failure, and even neurodevelopmental impairment (10, 15, 17).

Despite years of research on NEC, the pathomechanisms remain elusive. Most data suggests that the pathogenesis is multifactorial and involves (1) immaturity of the newborn’s gut and immune system (2), intestinal hyperinflammation, and (3) ischemia-reperfusion injury (4, 18). As such, bacterial exposure of an immature intestinal tract postpartum reportedly results in an exaggerated inflammation driven by the newborns’ immune system. While term-born neonates seem to control and downregulate their immune response to allow proper bacterial colonization, preterm-born neonates are not yet capable of this process (19). In addition, preterm-born neonates’ immature intestinal barrier may predispose them to develop NEC. This is due to a decreased mucus layer, low IgA synthesis, diminished intestinal peristalsis, and impaired tight junction activity (5, 12, 19). The increased expression of toll-like receptor 4 (TLR-4) on enterocytes in the gut of preterm-born neonates, when compared with term-born neonates, supports the role of intestinal barrier impairment and hyperinflammation in NEC development (20–22). This is paramount, as intestinal TLR-4 activation can lead to exaggerated enterocyte apoptosis, decreased restitution, bacterial enterocyte internalization and consequent microbial translocation (20, 23–26). Therefore, as part of NEC’s pathogenesis, the upregulation of TLR-4 may explain the damaging effects of physiological exposure to lipopolysaccharides (LPS) at the epithelial layer (12). What is more, binding of LPS to TLR-4 of macrophages causes a surge in the production of various proinflammatory chemokines and cytokines, which attract immune cells, including neutrophils, to the site of inflammation (23, 27). This surge can further exacerbate the damage caused to the epithelial layer of the neonatal intestine and cascade beyond the epithelium (23, 28).

Neutrophils are short-lived leukocytes of the innate immune system. As polymorphonuclear cells, they are highly maneuverable, meaning the multi-lobe nucleus helps them squeeze through small pores of the extracellular matrix and between cells (29, 30). As a result, they can migrate from the peripheral vascular system to virtually all tissues. As such, they constitute first-line responders of the innate immune system and can combat infectious pathogens using phagocytosis, degranulation, oxidative bursts, and the formation of neutrophil extracellular traps (NETs). NETs are web-like DNA structures decorated with a variety of antimicrobial agents, including toxic histones, antimicrobial peptides, and bactericidal proteins, each contributing to the neutrophil’s ability to neutralize pathogens (29, 31, 32). However, not only do NETs immobilize, inactivate, and kill pathogens, but they also trigger the production of further inflammatory mediators and proteases (33). As a drawback, the latter further damages inflamed sites and consequently promotes local tissue destruction. Moreover, neutrophils and NETs can produce inflammatory attractants and mediators that damage connective tissue and healthy cells (34). Thus, it is hypothesized that overly activated neutrophils exacerbate the local hyperinflammation reaction and drive the etiopathogenesis of NEC (35). This hypothesis was supported by findings observed in a murine model for NEC (36–38). However, as findings from animal models cannot directly be translated to humans (39), human organoid cultures might help address these limitations and overcome the gap between animal and human research.

Organoids are *in vitro* 3D organ systems formed from either pluripotent or organ-specific adult stem cells (40). They mimic native tissue and can differentiate into different cell types when supplied with appropriate growth factors (41). As such, organoid models have enabled research in tissue biology, development, and regeneration, as well as disease modelling, host-pathogen, and cell-cell interactions (40). Intestinal organoids, in particular, permit the examination of intestinal function, which can be used for drug discovery screening and to model disease phenotypes like Hirschsprung’s disease, short bowel syndrome, cystic fibrosis and NEC (42).

As access to human NEC samples is finite and murine NEC models have limitations, intestinal organoids may offer an excellent opportunity to study and understand the pathogenesis of NEC and ultimately provide a platform to test treatment options (43, 44). Thus, our study aimed to develop a NEC organoid model by employing human intestinal organoids grown from organ-specific adult stem cells harvested from intestinal samples of patients with NEC. To examine the role of neutrophils in NEC, we designed a coculture model using freshly isolated human neutrophils. We hypothesized that organoids from NEC differ from those of healthy controls (CO), and that these differences can be enhanced by adding (1) LPS and (2) LPS combined with neutrophils, as previously observed in animal models. Furthermore, organoid-based studies fulfil the three R principles of animal experimentation: replacement, reduction, and refinement (45, 46).

## 2 Methods

### 2.1 Neutrophil survival and activation

#### 2.1.1 Experimental setup

To assess whether neutrophils are affected by the growth medium used in the organoid culture (47), neutrophil survival and activation using flow cytometry were evaluated. Analyses compared (1) neutrophil survival measured by propidium iodide (PI)/Annexin-V (Annexin) staining (48), and (2) neutrophil activation, by staining of CD66b (secondary granules, neutrophil specific) (49) and CD11b (adhesion) (50, 51). Moreover, neutrophils were cultured in (1) IntestiCult Organoid Differentiation Medium Human (ODM; StemCell Technologies, Vancouver, Canada, Cat. 100-0214) with 100 U/ml Penicillin Streptomycin (P/S; ThermoFisher Scientific, Waltham, MA, USA, Cat. 15140-122) and 5 µm DAPT Notch Pathway Inhibitor (DAPT; StemCell Technologies, Vancouver, Canada, Cat. 72080), or (2) our laboratory’s standard neutrophil medium, i.e., Roswell Park Memorial Institute 1640 Medium (RPMI; ThermoFisher Scientific, Cat. A7030), with 1% bovine serum albumin (BSA; Sigma Aldrich, Saint Louis, MO, USA, Cat. 21875-034). To assess whether LPS from Escherichia coli O55:B5 (Sigma Aldrich, Saint Louis, MO, USA, Cat. L2880) affects neutrophils’ survival and activation, additional analyses were performed in the presence of LPS.

#### 2.1.2 Isolation of neutrophils

Neutrophils were isolated from human peripheral blood samples provided by healthy volunteers, using the MACSxpress^®^ Whole Blood Neutrophil Isolation Kit, human (Miltenyi Biotech, Bergisch Gladbach, Germany, Cat. 130-104-343), according to manufacturers’ instructions: In total, 8 ml of whole blood (EDTA) was mixed with 4 ml of the MACSxpress Isolation Mix (buffers A and B, magnetic beads), from the MACSxpress^®^ Whole Blood Neutrophil Isolation Kit, human (Miltenyi Biotech, Bergisch Gladbach, Germany, Cat. 130-104-343), followed by an incubation period of 5 min at room temperature in the MACSmix Tube Rotator (Miltenyi Biotech, Cat. 130-090753) at 12 rpm. The neutrophils were then separated using the MACSxpress^®^ Separator (Miltenyi Biotech, Cat. 130-098-308) over 15 min at room temperature. The supernatant containing the neutrophils was centrifuged at 300xg for 10 min at 4°C. After centrifugation, any remaining erythrocytes were lysed by resuspension in 2 ml of distilled H_2_O, followed by the addition of 0.7 ml of 0.6 M KCL solution (Th. Geyer, Renningen, Germany, Cat. 1632.0500). The cells were then washed with PBS and centrifuged at 250xg for 7 min at 4°C. The pelleted neutrophils were resuspended in the assay media.

Neutrophils were quantified by hemocytometry. Vital cells were identified by 0.4% Trypan Blue exclusion (ThermoFisher Scientific, Cat. 15250-061). Based on cell count, neutrophils were diluted to 1*10^6^ cells/ml for all subsequent experiments. The purity of the neutrophils was verified by flow cytometry, using antibodies against CD15 (48) (FITC anti-human CD15, mAb, Clone: HI98, mouse IgM, Cat. 301904) and CD16 (50) (PerCP anti-human CD16, mAb, Clone: 3G8, mouse IgG1, Cat. 302030) (Biolegend, San Diego, CA, USA).

#### 2.1.3 Short-term neutrophil culture

After neutrophils were resuspended in either (1) ODM or (2) RPMI medium (containing 1% BSA), cells either remained untreated or were treated with 200 µg/ml LPS. All cells were cultured in flat-bottom 48-well cell culture plates at 37°C and 5% CO_2_. Flow cytometry (see 1.4) was performed at 0, 3, 6, 12, 24, 48, and 72 h.

#### 2.1.4 Flow cytometry

Neutrophils were transferred to 5 ml tubes, and adherent neutrophils were gently detached using Accutase Cell Detachment Solution (Capricorn Scientific, Ebsdorfergrund, Hessen, Germany, Cat. ACC-1B) for 30 min at 37°C. Then cells were washed with 1X Dulbecco’s phosphate-buffered saline (PBS; ThermoFisher Scientific, Cat. 14190-094). Next, neutrophils were either stained with (1) PI (Becton Dickinson and Company, Cat. 51-66211E, dilution: 1:20 in Annexin Binding Buffer (10X Annexin Binding Buffer, Becton Dickinson and Company, Cat. 5166121E) diluted 1:10 in Aqua dest.) and Annexin (Cat. 556419, dilution: 1:50 in Annexin Binding Buffer) for detection of apoptosis and necrosis, or (2) antibodies against CD66b (APC anti-human CD66b, Reafinity™, mAb, Clone: REA306, human IgG1, Milenyi Biotec, Bergisch Gladbach, Germany, Cat. 130-117-692, dilution: 1:50 in autoMACS Rinsing Solution (Milenyi Biotec, Cat. 130-091-222) with 0,5% BSA (MACS Staining Buffer) and CD11b (Vioblue anti-human CD11b, Reafinity™, mAb, Clone: REA713, human IgG1, Milenyi Biotec, Cat. 130-110-554, dilution: 1:50 in MACS Staining Buffer) for detection of neutrophil activation. Staining was performed according to the manufacturer’s protocols. Analysis was carried out using FACSCanto™II and FACSDiva™ 8.0.2 (Becton Dickinson and Company). Each condition was taken in technical duplicates and replicated three times.

### 2.2 Organoid cultivation and maintenance

#### 2.2.1 Human intestinal sample collection

Human intestinal samples were collected at the Department of Pediatric Surgery of the University Medical Center Hamburg-Eppendorf and the Altona Children’s Hospital Hamburg.

For NEC organoids, intestinal tissue samples were obtained from patients suffering from severe NEC during emergency bowel resection surgery (n=7). Age-matched intestinal CO organoids were grown from non-inflamed intestinal samples of patients undergoing corrective surgery for congenital gastrointestinal abnormalities (i.e. intestinal atresia surgery) or ileo- or colostomy procedures (n=7). Harvested samples included both ileum and colon, which were case-control matched. Detailed clinical and demographic data for both groups, including sex, age at surgery, birth weight, resection location (ileum vs. colon), and presence of hemodynamically relevant cardiac comorbidities, are provided in **Table 1**. Statistical analysis confirmed no significant differences between the two groups for any of these characteristics.

**Table 1:** Clinical and demographic data of the patients used for organoid development.

Characteristic	NEC (n=7)	Control (n=7)	P value
Sex (M/F)	3/4	3/4	>0.9999
Age (d)	226 ± 47	224 ± 53	0.4723
Age (m) at Surgery	3.5 ± 4.8	4.5 ± 2.5	0.647
Weight at Birth (g)	1754 ± 1413	1752 ± 970.7	0.4992
Ileum/Colon Resection	4/3	4/3	>0.9999
Hemodynamically relevant cardial comorbidities (Y/N)	2/5	3/4	>0.9999

Data are presented as number or mean ± SD. Statistical analyses were performed using the unpaired t-test or Fisher’s exact test.

#### 2.2.2 Isolation of intestinal crypts

To obtain the best yield of intestinal stem cells, samples were directly placed in Iscove’s Modified Dulbecco’s medium (IMDM; ThermoFisher Scientific, Cat. 12440053) containing 20% fetal bovine serum (FBS; ThermoFisher Scientific, Cat. 10500-064) and 1% P/S and processed within 24 h after surgery.

Preparation involved a longitudinal dissection of the sample to uncover the intestinal mucosa containing the epithelial cell layer. Following the dissection, the sample was rinsed using PBS to remove stool and mucus. Next, using scissors the epithelial cell layer was separated from the underlying connective tissue and muscle layer. The epithelial cell layer was cut into 0.1 cm^2^ pieces and incubated in Iscove’s Modified Dulbecco’s Medium (ThermoFisher Scientific, Cat. 21980-032) supplemented with 5 mM ethylenediaminetetraacetic acid (0.5 M UltraPure, Life Technologies, California, USA, Cat. 15575-038), 2 mM 1,4-dithiothreitol (Sigma-Aldrich, Missouri, USA, Cat. D9779-5G), 5% FBS and 1% P/S for 40 min at 4°C on a plate shaker. This procedure detached the intestinal crypts - harboring the intestinal stem cells - from the epithelial cell layer. To obtain single-cell solutions, aggregates were removed with a 70 µm cell strainer. The solution was centrifuged for 10 min at 500xg and 4°C. Cells were resuspended in 10 ml of Advanced DMEM/F12 (ThermoFisher Scientific, Cat. 12634-010), supplemented with 1% of HEPES (Sigma Aldrich, Missouri, USA, Cat. H3537-100ML), 1% GlutaMAX (ThermoFisher Scientific, Cat. 35005-061), and 1% of P/S (further referred to as Adv. +++) and centrifuged for another 10 min at 500xg and 4°C. The cell pellet was suspended in up to 200 µl of Adv. +++ depending on organoid yield. 40 µl of Adv. +++ containing the cells were mixed with 80 µl of Matrigel Basement Membrane Matrix (Matrigel; Corning, New York, USA, Cat. 356321). Four 30 µl drops of the mixture were seeded onto prewarmed cell culture plates and dried for 30 min at 37°C. Then 500 µl (24-well plates) or 1000 µl (12-well plates) of warm IntestiCult Organoid Growth Medium Human (OGM; Stemcell Technologies, Cat. 06010) containing 100 units/ml of P/S, and, until first passaging, also 10 µM Y27632 ROCK-Pathway Inhibitor (Stemcell Technologies, Cat. 73203), was added to each well.

#### 2.2.3 Maintenance of organoid cultures

**Figure 1:**
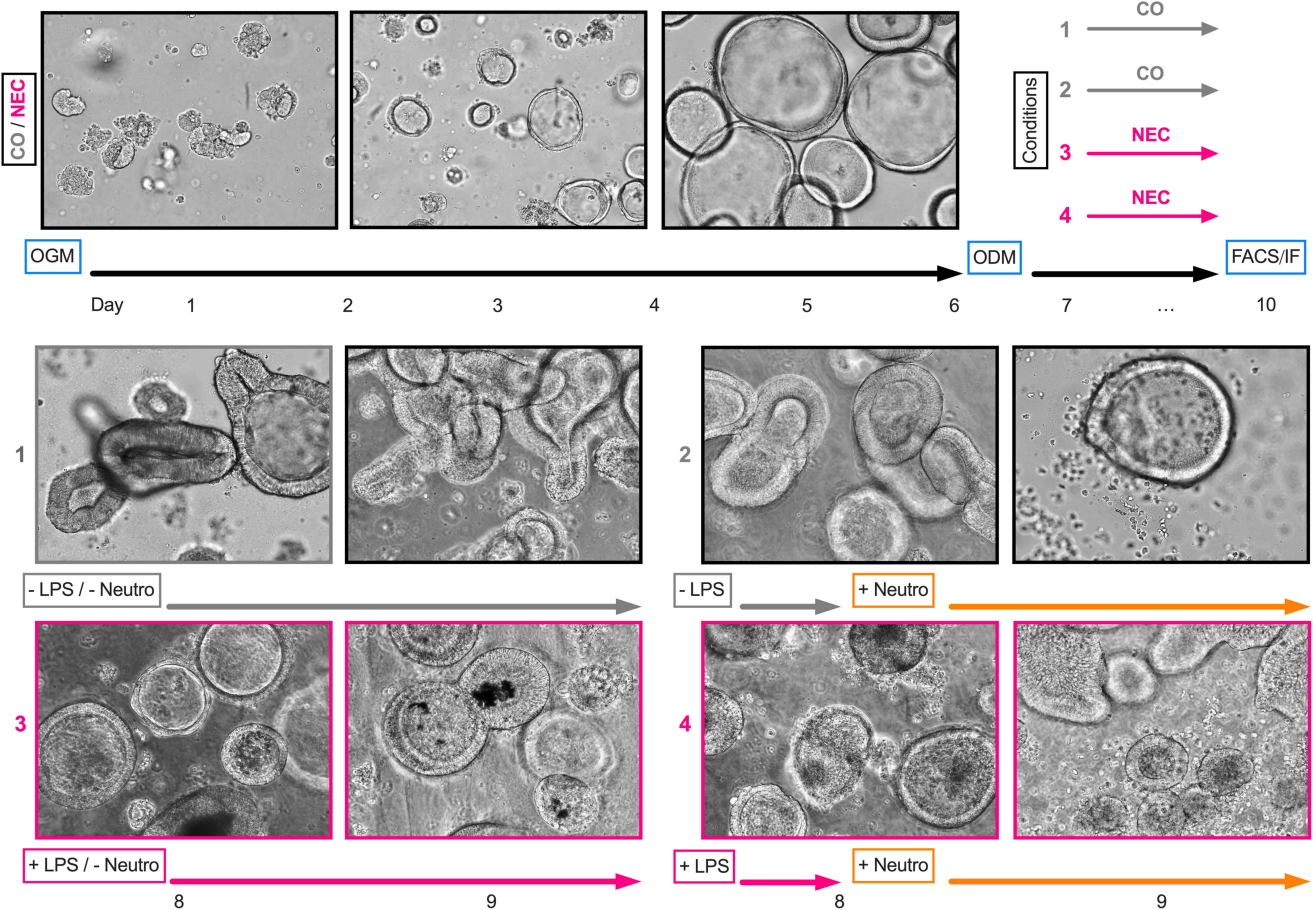
Experimental design and light microscopy images. After passaging organoids for a minimum of three times using an organoid growth medium (OGM), organoid differentiation was initiated on day six using an organoid differentiation medium (ODM). For lipopolysaccharide (LPS) stimulation, LPS was added to the medium on day seven and replaced after 24 h. For cocultures, neutrophils (Neutro) were added to the medium on day eight. On day nine, cultures were further processed for fluorescence-activated cell sorting analysis or immunofluorescence staining. The light microscopy images depict one sample (ileum) obtained from a NEC patient across the course of the experimental design.

Organoids were cultured at 37°C and 5% CO_2_. OGM was changed every 2 to 3 days, containing ROCK-Pathway Inhibitor until first passaging on day 7. Organoids were passaged every 7 days to maintain a healthy culture and to remove debris, dead cells, and shredding large organoids (**Figure 1**). To passage the organoids, Matrigel drops containing the growing spheroids were dissolved in ice-cold Adv. +++, transferred into a centrifugation tube, and centrifuged for 5 min at 300xg and 4°C. Then, 1 ml of ice-cold Adv. +++ was added to the pellet and organoids were disrupted by repeated pipetting. The dissociated organoids were centrifuged for 5 min at 400xg and 4°C. The pellet was resuspended in up to 300 µl of ice-cold Adv. +++ and was mixed with Matrigel in a ratio of 1:2. Drops of 30 µl per well were then seeded onto prewarmed cell culture well plates and dried in the incubator for 30 min before 500 µl of OGM was added to the wells. Organoid development was monitored daily using a light microscope (Leica DM IL LED, Type 11 090 137 001) (**Figure 1**).

### 2.3 LPS stimulated NEC organoid model

Organoids were passaged at least three times before being used in any experiments. For experiments, the intestinal organoids were sown separately from the stock culture to prevent cross-contamination. Before organoids were used for experimental purposes, their differentiation was induced by a medium change from OGM to ODM on day 6 (**Figure 1**). In line with animal studies for NEC (36, 43) and other NEC intestinal organoid models (44, 52), NEC was induced by adding LPS to differentiated organoids. The cultures were incubated with LPS for 48 h in total, renewing the LPS-containing medium after 24 h (**Figure 1**).

### 2.4 Coculture of NEC organoids and neutrophils

Organoids used for neutrophil cocultures were generated as described above. After 24 h of incubation with (1) ODM or (2) ODM+LPS, freshly isolated human neutrophils (section 1.1) were added to the organoid cultures. After isolation, neutrophils were suspended in ODM and, depending on the group, 200 µg/ml LPS (44). The suspension was then added to the culture pellets (500 µl/well). After 24 h, cells were assessed via flow cytometric or immunofluorescence (IF) analyses.

To distinguish neutrophils from organoid cells, neutrophils were stained with a CSFE Cell Labeling Kit (Abcam, Cambridge, UK, Cat. Ab113853) before being added to the organoids. After neutrophil isolation, neutrophils were resuspended in CSFE Cell Labeling Kit, diluted in PBS (5 µM CSFE Cell Labeling Kit) and incubated at room temperature for 15 min. Lastly, neutrophils were washed with PBS with 1% BSA to remove non-incorporated dye and centrifuged for 2 min at 300xg. The neutrophil pellet was then suspended in ODM+/-LPS at 1*10^6^ cells/ml.

### 2.5 Flow cytometry

#### 2.5.1 Organoids without neutrophils

In preparation for flow cytometric analyses, organoid pellets were washed with Adv. +++ to dissolve the Matrigel, centrifuged for 5 min at 300xg and 4°C, and dissociated using TrypLE Express (ThermoFisher Scientific, Cat. 12605-028). In TrypLE Express, cells were incubated at 37°C for 3 min before being mechanically broken up by pipetting (section 2.3) to obtain a single-cell suspension. Unspecific antibody binding sites were blocked through incubation with FBS (10%) for 20 min at 4°C. Cells were then washed with PBS, and antibodies were added: Epithelial cells were labelled with APC-Cy7 Zombie NIR Fixable Viability Dye (Zombie Assay; BioLegend, Cat. 423105, dilution: 1:2500 in PBS) and extracellular TLR-4 was stained using a Pacific Blue labelled TLR-4 antibody (BV421 mouse anti-human TLR-4, mAb, Clone: TF901, mouse IgG1 Becton Dickinson and Company, Cat. 564401, dilution: 1:40 in PBS). Following an incubation period of 30 min at 4°C in the dark, cells were washed with PBS and fixed with 20% BD CellFIX (Becton Dickinson and Company, Cat. 340181) in PBS. Slides were analyzed using a LSR Fortessa Flow Cytometer (BD Biosciences) with FACSDiva™8.0.2 software. Gates were set using unstained and fluorescence-minus-one (FMO) controls.

#### 2.5.2 Neutrophil cultures

To assess TLR-4 expression neutrophils were cultured at 37°C and 5% CO_2_. Cells were re-suspended and cultured in either ODM or ODM+LPS, identical to organoid cultures (5.1) and cocultures (5.3). Analysis by flow cytometry was carried out after 24 h to survey TLR-4 expression, viability, and activation. Before flow cytometry, neutrophils were stained with CFSE (4.2). Staining and analysis of neutrophil-only cultures were treated like organoid-only or cocultures (5.1, 5.3). Unstained, single-stained, and FMO controls were used to gate various markers and compensate for unspecific antibody binding.

#### 2.5.3 Neutrophil organoid coculture

Organoids to be cocultured with neutrophils were prepared as described in section 5.1. To assess neutrophil activity using flow cytometry, anti-CD11b (PE anti-human CD11b, Reafinity™, mAb, Clone: REA713, human IgG1, Miltenyi Biotec, Cat. 130-110-553, dilution: 1:100 in PBS) was added to the antibody mixture (containing the Zombie Assay and TLR-4 Antibody). To identify neutrophil apoptosis a Zombie NIR Dye was employed, which permanently dyes compromised cell membranes and therefore marks dying cells. Furthermore, as described in section 4.2, neutrophils were labelled with CSFE Cell Labeling Kit before addition to the organoid cultures. Single-stained and unstained controls were employed to calculate compensation, and unstained and FMO controls to adjust gates.

### 2.6 Immunofluorescence

For IF analyses, organoids were harvested in Matrigel as described above and seeded on round cover glasses, prewarmed in an incubator for 24 h. Cultures for IF were maintained in OGM for 5 days before cell differentiation was initiated by replacing OGM with ODM for 72 h. After 24 h of ODM, the LPS organoid cultures were treated with 200 µg/ml LPS for 48 h.

Staining of organoid-only cultures commenced after either (1) 72 h of ODM, or (2) 24 h of ODM, followed by 48 h of ODM with LPS. For cocultures, neutrophils were added in the last 24 h of treatment, so that samples were fixed for IF after 24 h of coculture.

#### 2.6.1 Immunofluorescence of organoids

Organoids were fixed on round cover glasses (see previous section) with Paraformaldehyde 4% in PBS (Morphisto GmbH, Offenbach am Main Germany, Cat. 11762.01000) at room temperature for one hour. This step dissolved the Matrigel completely. Fixed samples were washed twice in PBS before being permeabilized with 0.2% Triton™ X-100 (Sigma Aldrich, Saint Louis, MO, USA, Cat. T8787) in PBS. Following 60 min of incubation and washing, unspecific antibody binding sites were blocked using 5% BSA in PBS (Blocking Buffer - BB) for one hour, and cultures were stained with E-cadherin antibody (E-Cad; pAb, rabbit IgG, GeneTex, Irvine, California, US, Cat. GTX100443, dilution: 1:500 in BB). One sample per group served as Isotype control (Rabbit IgG, pAb, Abcam, Cat. Ab37415, dilution: 1:2000 in BB). All samples were incubated in antibody buffer overnight at 4°C and washed five times with PBS. The secondary antibody Alexa Flour^®^ 647-conjugated AffiniPure Donkey Anti-Rabbit IgG (Jackson ImmunoResearch, Ely, Cambridgeshire, UK, Cat. 711-605-152; dilution: 1:200 in BB) was added for one hour at room temperature in darkness. Next, samples were washed five times before staining all samples with 1 µg/ml DAPI (Carl Roth, Karlsruhe, Germany, Cat. 6355.1) in PBS for 5 min. Afterwards, samples were washed in PBS and mounted using 20 µl of Fluoromount G (SouthernBiotech, Birmingham, Alabama, US, Cat. 0100-01).

#### 2.6.2 Immunofluorescence of neutrophil organoid cocultures

As neutrophils had already been stained with the CFSE Labeling Kit (section 4.2), staining for IF in coculture was conducted similarly to the organoids-only culture (section 6.1). In addition to the E-cadherin antibody used in the organoid-only IF, staining for the neutrophil marker CD15 with antibodies against CD15 (anti-CD15 antibody, mAb, Clone: 153B, mouse IgM, Abcam, Cat. Ab241552, dilution: 1:250 in BB) was also performed. A murine IgM Isotype Control (Clone: 11E10, ThermoFisher Scientific, Cat. 14-4752-82, dilution: 1:125 in BB) served as negative control. As a secondary antibody, Cy™3 AffiniPure Donkey Anti-Mouse IgG (Jackson ImmunoResearch, Cat. 715-165-150; 1:200 in BB), was employed. Isotype controls were used to determine the specific binding of antibodies to E-cadherin (organoids) and CD15 (neutrophils).

#### 2.6.3 Microscopy

Imaging was conducted with a confocal microscope LEICA TCS SP5 using (1) 1.4*60/63 OIL to study the organoids’ morphology and (2) 0.75*20 IMM as an overview. Images were taken and digitalized with LEICA LAS software. Images of isotype controls were taken using the same laser power as the specific antibodies. The color of E-Cad (Alexa Flour 647 as far-red dye) was changed from red to green using the software.

#### 2.6.4 Scoring

An organoid NEC-Status Score was designed to evaluate organoid morphology based on an adapted version of the Chiu-Score (Chiu et al., 1970) designed to assess intestinal damage (53). The score involves (1) number of mitoses (0 – many; 1 – moderate; 2 – few; 3 – none) (2), cell border status (0 – borders intact; 1 – minor damage; 2 – moderate damage; 3 – major damage) (3), cell nuclei’s health status (0 – nuclei intact; 1 – minor damage; 2 – moderate damage; 3 – major damage), and (4) cell detritus (0 – none; 1 – minor amount; 2 – moderate amount; 3 – major amount). The 12 points were translated into a NEC-Status Score as follows:

NEC-Status Twelve-Point Scale:

- 0 - 2 = no signs of barrier disruption
- 3 - 5 = minor barrier disruption
- 6 - 9 = moderate barrier disruption
- 10 - 12 = major barrier disruption

In images involving neutrophils and intestinal organoids, the neutrophil count was scored as follows: none (0), few (1), medium (2), or many (3) neutrophils. Only neutrophils that had migrated through the Matrigel were counted, as neutrophils in the supernatant were washed away before fixing the Matrigel drop. Scoring was conducted by two independent reviewers blinded to the groups.

### 2.7 Human samples and ethics approval

Intestinal tissue samples used for organoid generation were obtained from NEC patients and age-matched CO as described in section 2.1. Peripheral blood for neutrophil isolation was collected from healthy adult volunteers under informed written consent (see section 1.2).

All procedures involving human tissue and blood samples were approved by the ethics committee of the Freie Hansestadt Hamburg Medical Association (Ärztekammer Hamburg) under protocol number PV5251. Written informed consent was obtained from all legal guardians or adult donors in accordance with the Declaration of Helsinki.

### 2.8 Statistics

Statistical analyses were conducted using GraphPad Prism 9 (GraphPad, CA, USA). Differences between groups were calculated with an ANOVA test. As the experiments involving neutrophil survival and activation resulted in data with repeated measurements, a repeated measures ANOVA with the Geisser-Greenhouse correction and Tukey’s multiple comparison test were employed. Organoid and neutrophil coculture data (IF and flow cytometry) was analyzed using a repeated-measures ANOVA with Geisser-Greenhouse correction and Holm-Sidak multiple comparisons test. Clinical and demographic differences between NEC and controls were assessed with unpaired t-tests or the Fisher’s exact test. Data are presented as mean ± standard deviation (SD). The level of significance was set at 0.05.

All authors had access to the study data and have reviewed and approved the final manuscript. Data, analytic methods, and study materials will be made available to other researchers upon request.

## 3 Results

### 3.1 Neutrophil activation and survival in organoid differentiation medium

To evaluate whether the organoid differentiation medium (ODM) affects neutrophil survival and activation, neutrophils were cultured for up to 72 h in our standard neutrophil medium (RPMI) or ODM with or without LPS). These analyses were crucial in determining the optimal length of the incubation period of the organoids with neutrophils.

**Figure 2:**
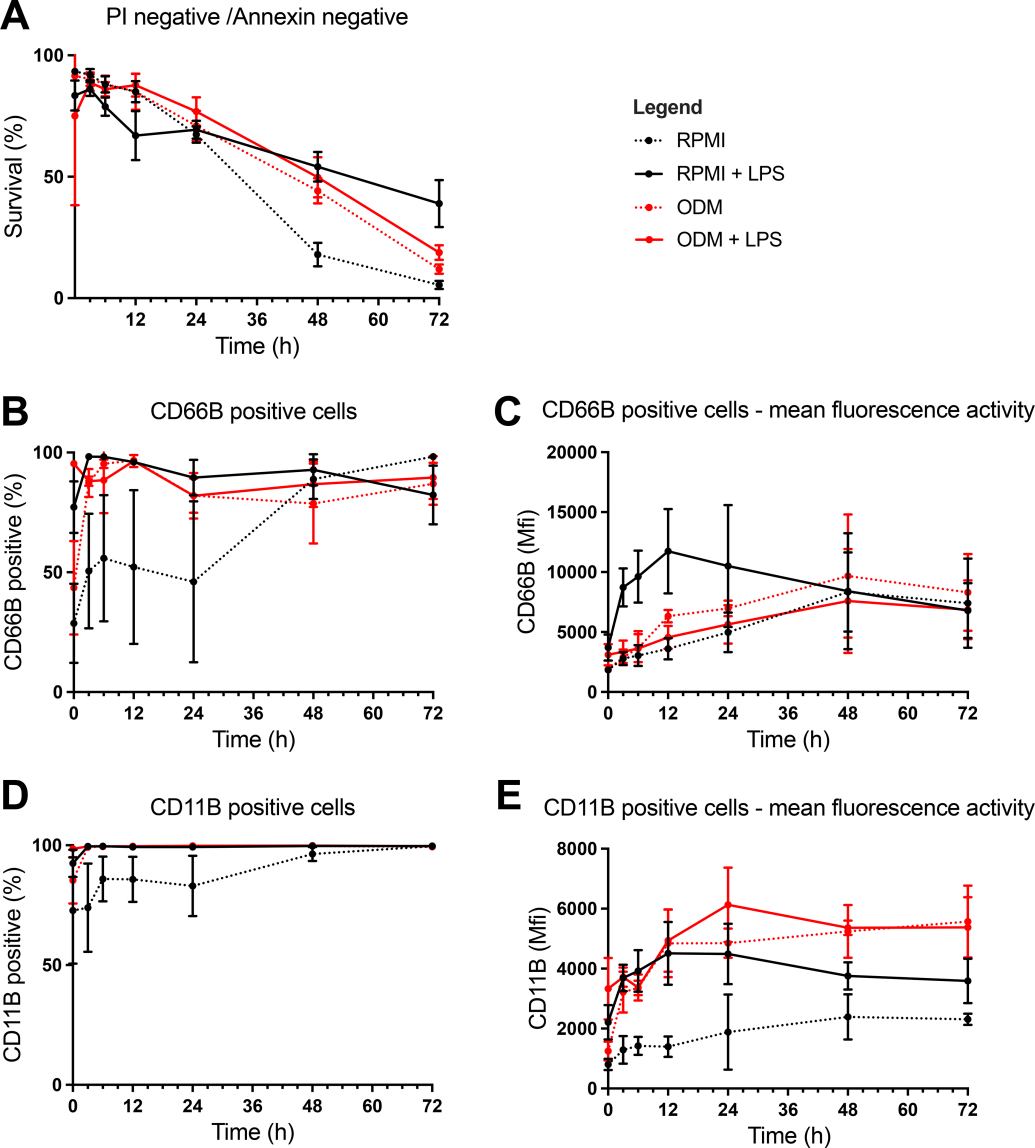
Assessment of neutrophil survival and activation using analysis by flow cytometry. Flow cytometry was performed to assess (1) neutrophil survival using propidium iodide (PI) and Annexin-V staining, and (2) activation using anti-CD66b and anti-CD11b antibodies, in the following conditions (1): Organoid differentiation medium (ODM) (2), Roswell Park Memorial Institute 1640 medium (RPMI) (3), ODM + lipopolysaccharide (LPS), and (4) RPMI + LPS. **(A)** Neutrophil survival was assessed at during multiple timepoints up until 72 (h) Survival rates decreased steadily until 24 h in all groups with no significant differences between groups. After 24 h, neutrophils in RPMI only showed significantly lower survival rates, suggesting a detrimental effect of RPMI alone on neutrophil viability. At 72 h, increased survival of neutrophils in LPS was observed. **(B, D)** Neutrophil activation was analyzed by CD66b **(B)** and CD11b **(D)** expression for 72 h at multiple timepoints. Activation markers (CD66b and CD11b) increased across all conditions at 24 h, with neutrophils in RPMI only showing similar activation levels to other conditions after 48 (h). This suggests a delayed activation in RPMI compared to other groups. **(C, E)** CD66b **(C)** and CD11b **(E)** signal intensity were measured at multiple time points up to 72 (h) CD66b signal intensity rose in (1) RPMI (2), ODM, and (3) ODM+LPS, while the signal intensity in RPMI+LPS solely rose within the first 12 h, upon which a decrease was seen **(C)**. CD11b intensity increased over time and plateaued after 12 h, with RPMI showing a less pronounced increase, indicating delayed activation **(E)**.

Neutrophil survival decreased steadily over time in all conditions after being cultured for 24 h, regardless of the condition (**Figure 2A**). Moreover, at 24 h there were no significant differences between groups. However, by 48 h, neutrophils cultured in RPMI alone showed significantly lower survival compared to those in RPMI + LPS (18% vs. 54%, p<0.0001), ODM (18% vs. 44%, p<0.0001), and ODM + LPS (18% vs. 50%, p=0.0002). At 72 h, LPS treatment significantly prolonged survival in both RPMI (5% (without LPS) vs. 39% (with LPS), p=0.0012) and ODM (12% (without LPS) vs. 19% (with LPS, p=0.0058).

Neutrophil activation (measures as CD66b+ neutrophil frequencies) increased in all conditions by 24 h (RPMI 46%, RPMI + LPS 90%, ODM 82%, ODM + LPS 82%) (**Figure 2B**). At 3 h, CD66b+ frequencies were significantly higher in RPMI + LPS (98%), ODM (87%), and ODM + LPS (88%) compared to RPMI alone (51%, p<0.05 for all comparisons). The same trend was seen for CD11b, yet without reaching significance (**Figure 2D**).

With respect to CD66b signal intensity, it continued to rise over time until 48 h, particularly in RPMI + LPS cultures (**Figure 2C**). CD11b signal intensity also increased over the first 12 h, plateauing afterward (**Figure 2E**). Neutrophils in RPMI displayed lower CD11b expression than those in RPMI + LPS (1642 vs. 3738, p<0.0001), ODM (1642 vs. 4048, p<0.0001), and ODM + LPS (1642 vs. 4603, p<0.0001). CD11b signal was also significantly higher in ODM + LPS than in RPMI + LPS (4603 vs. 3738, p=0.0058).

Based on these results, a 24-hour coculture period was selected for neutrophil-organoid experiments to maintain optimal neutrophil viability and activation.

Based on these results, a 24 h co-culture period was selected for neutrophil-organoids experiments to maintain optimal neutrophil viability and activation. On average, neutrophils showed the highest survival and activation levels within the first 24 h of incubation in ODM (with or without LPS) in monoculture. While viability was highest within the first 6 h, our data show that neutrophils retained >50% viability and exhibited stimulus-specific activation up to 24 h, supporting this time frame as a biologically relevant window for studying neutrophil-epithelial interactions. Moreover, a 24 h period reflects the dynamic and evolving nature of inflammatory processes in NEC, which typically develop over several hours to days.

### 3.2 Organoid analyses in coculture with neutrophils

#### 3.2.1 Epithelial apoptosis and TLR-4 expression in organoids

To assess the effect of LPS treatment and the addition of neutrophils on intestinal organoids, the inflammatory response of epithelial cells in organoids, as determined by TLR-4 expression and apoptosis, was measured.

**Figure 3:**
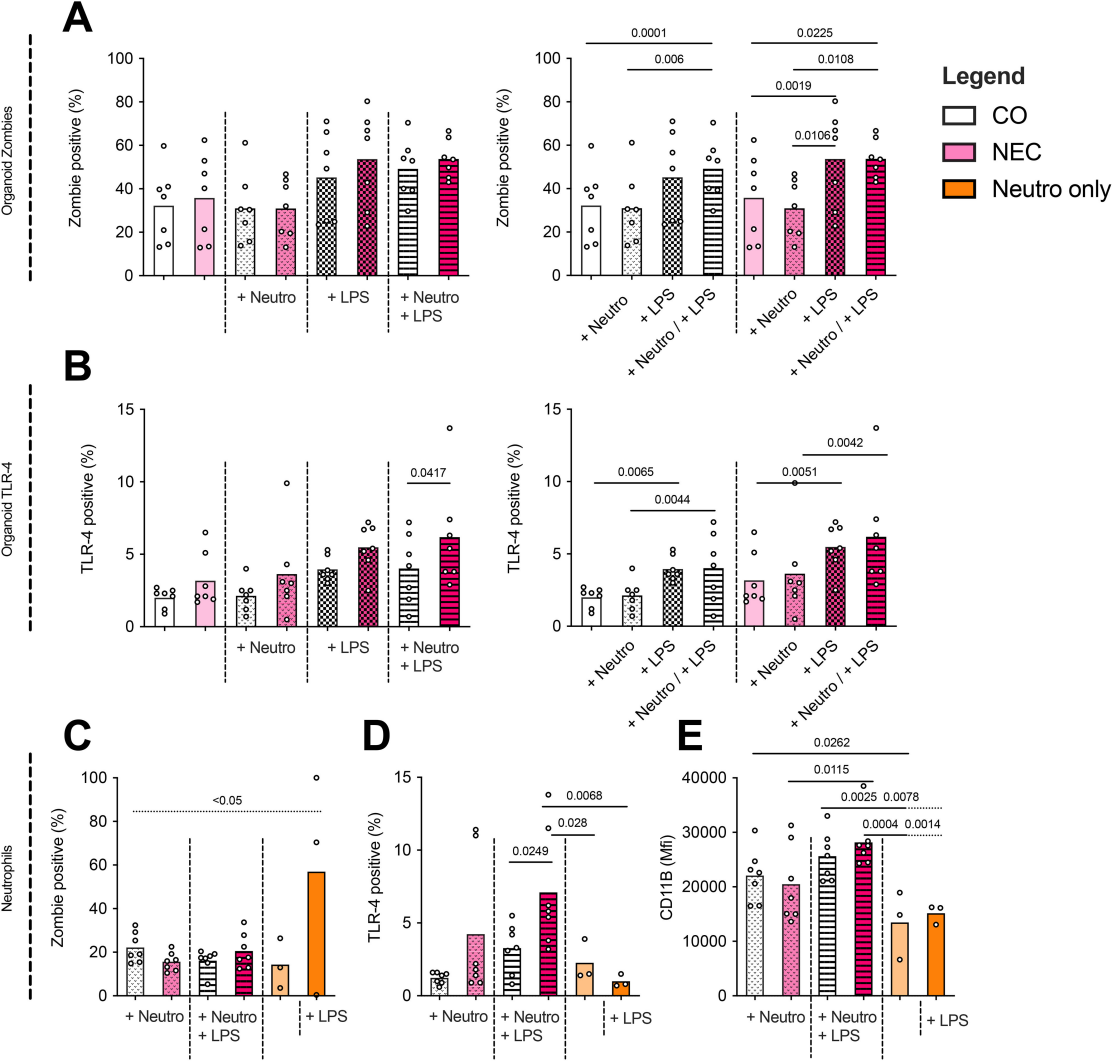
Analyses by flow cytometry of neutrophil–organoid cocultures. **(A)** NEC and control (CO) organoid apoptosis (Zombie Aqua staining) increased non-significantly across most conditions, with a significant rise observed in NEC organoids upon LPS stimulation. Additionally, significant increases in apoptosis were detected when comparing NEC or CO organoids alone versus their respective LPS + neutrophil cocultures. **(B)** TLR-4 expression in organoids was significantly elevated in all conditions following LPS stimulation or coculture with LPS-stimulated neutrophils, with the pronounced effect observed in NEC organoids. **(C)** Neutrophils were cultured for 24 h under six conditions (1): in ODM alone (2), in ODM + LPS (3), with CO organoids (4), with CO organoids + LPS (5), with NEC organoids, and (6) with NEC organoids + LPS. LPS stimulation in monoculture (group 2) significantly increased neutrophil apoptosis compared to the unstimulated control (group 1). In contrast, apoptosis rates were significantly reduced in all coculture conditions (groups 3–6) compared to the LPS-only monoculture, indicating a potential protective effect mediated by epithelial interaction. **(D)** Neutrophil TLR-4 expression showed a non-significant increase when cocultured with organoids, but was significantly higher in LPS-stimulated cocultures with NEC versus CO organoids. **(E)** Neutrophil activation, as measured by CD11b signal intensity, increased significantly when neutrophils (+/– LPS) were cocultured with CO organoids compared to neutrophil monocultures (+/– LPS). This difference was also observed when comparing neutrophils cocultured with LPS-stimulated organoids versus LPS-stimulated neutrophils alone. No significant differences were found in neutrophil activation between CO and NEC cocultures (+/– LPS).

Zombie staining of epithelial cells in organoids revealed higher apoptosis in NEC organoids stimulated with LPS compared to untreated NEC organoids (53.69 ± 22.11% vs. 35.74 ± 19.91%, p=0.0019) (**Figure 3A**). There was no increase in apoptosis upon addition of neutrophils without LPS. When neutrophils and LPS were added, a significant increase in apoptosis was observed in both NEC (35.74 ± 19.91% vs. 53.74 ± 8.86%, p=0.0225) and CO (32.2 ± 16.89% vs. 49.14 ± 13.65%, p=0.0001) organoids compared to their respective unstimulated controls.

Cultures of NEC stimulated with LPS only showed a significantly increased TLR-4 expression compared to CO cultures with LPS when additionally stimulated with neutrophils (NEC + LPS + neutrophils mean 6.19+/-3.67%; CO + LPS + neutrophils mean 4.01+/-2.38%, p=0.0417). Overall, the highest TLR-4 expression was measured in NEC LPS neutrophil cocultures (mean 6.19+/-3.67%) as seen in **Figure 3B**.

All comparisons are visualized in both graphs in **Figures 3A, B**, which display the same dataset (zombie staining and TLR-4 staining respectively) in different groupings to aid interpretation. Significances for within-group comparisons are only depicted on the right graphs.

### 3.3 Neutrophil phenotype in coculture with organoids

To determine whether neutrophils interact functionally with epithelial cells of intestinal organoids (CO and NEC), we analyzed neutrophil apoptosis after a 24-hour coculture period, based on prior data showing optimal neutrophil survival and activation over this time course. The following six conditions were compared (1): neutrophils in ODM without organoids (control) (2), neutrophils in ODM with LPS but without organoids (control LPS) (3), neutrophils in ODM with CO organoids (4), neutrophils in ODM with CO organoids and LPS (5), neutrophils in ODM with NEC organoids, and (6) neutrophils in ODM with NEC organoids and LPS.

Zombie dye analysis of neutrophils revealed increased apoptosis in LPS-stimulated monocultures compared to cocultures with CO or NEC organoids (p<0.05 for all comparisons). Neutrophils cultured with LPS alone also showed significantly higher apoptosis than those without LPS in monoculture (p<0.05) (**Figure 3C**).

As shown in **Figure 3C**, neutrophils stimulated with LPS in monoculture (group 2) exhibited significantly higher apoptosis rates than unstimulated monocultures (group 1), confirming the pro-apoptotic effect of LPS. Importantly, neutrophils cocultured with organoids (groups 3–6) showed significantly reduced apoptosis compared to the LPS-only monoculture condition (group 2), regardless of whether LPS was present or whether the organoids were derived from CO or NEC tissue. This suggests a potential protective effect of epithelial interaction against LPS-induced neutrophil apoptosis.

TLR4 expression on neutrophils remained comparable after coculture with NEC organoids (mean 4.23 ± 4.79%) or CO organoids (mean 1.23 ± 0.39%). However, TLR4 expression significantly increased in neutrophils cocultured with NEC organoids following LPS stimulation (CO: 3.27 ± 1.70%; NEC: 7.10 ± 4.00%; *p* = 0.0249). This LPS-induced upregulation was not observed in neutrophils cultured without organoids (**Figure 3D**).

CD11b signal intensity on neutrophils remained unchanged in coculture with organoids compared to neutrophil monocultures. However, neutrophils cocultured with CO organoids exhibited a significant increase in CD11b intensity compared to monocultured neutrophils (CO + neutrophils: 22,057 ± 4,843; neutrophils alone: 13,469 ± 6,267; *p* = 0.0262). Following LPS stimulation, neutrophils cocultured with either NEC or CO organoids showed significantly higher CD11b signal intensity than neutrophils in monoculture, regardless of LPS treatment (NEC + LPS + neutrophils: 28,159 ± 4,829; CO + LPS + neutrophils: 25,629 ± 4,431; neutrophils: 13,469 ± 6,267; neutrophils + LPS: 15,144 ± 1,813; *p* < 0.05). Consistent with findings on neutrophil survival and TLR4 expression, LPS stimulation did not further enhance activation in neutrophils cocultured with CO organoids. Additionally, there were no significant differences in neutrophil activation between cocultures with CO versus NEC organoids, irrespective of LPS stimulation. Notably, a significant increase in CD11b intensity was observed when neutrophils were cocultured with NEC organoids and stimulated with LPS compared to unstimulated NEC cocultures (NEC + neutrophils: 20,498 ± 7,112; NEC + LPS + neutrophils: 28,159 ± 4,829; *p* = 0.0115) (**Figure 3E**)

Given the small sample size of the neutrophil only experiments, these findings should be interpreted as exploratory, and additional studies will be needed to confirm these effects in larger datasets.

### 3.4 NEC-like morphology and neutrophil recruitment in organoids (using immunofluorescence analyses)

**Figure 4:**
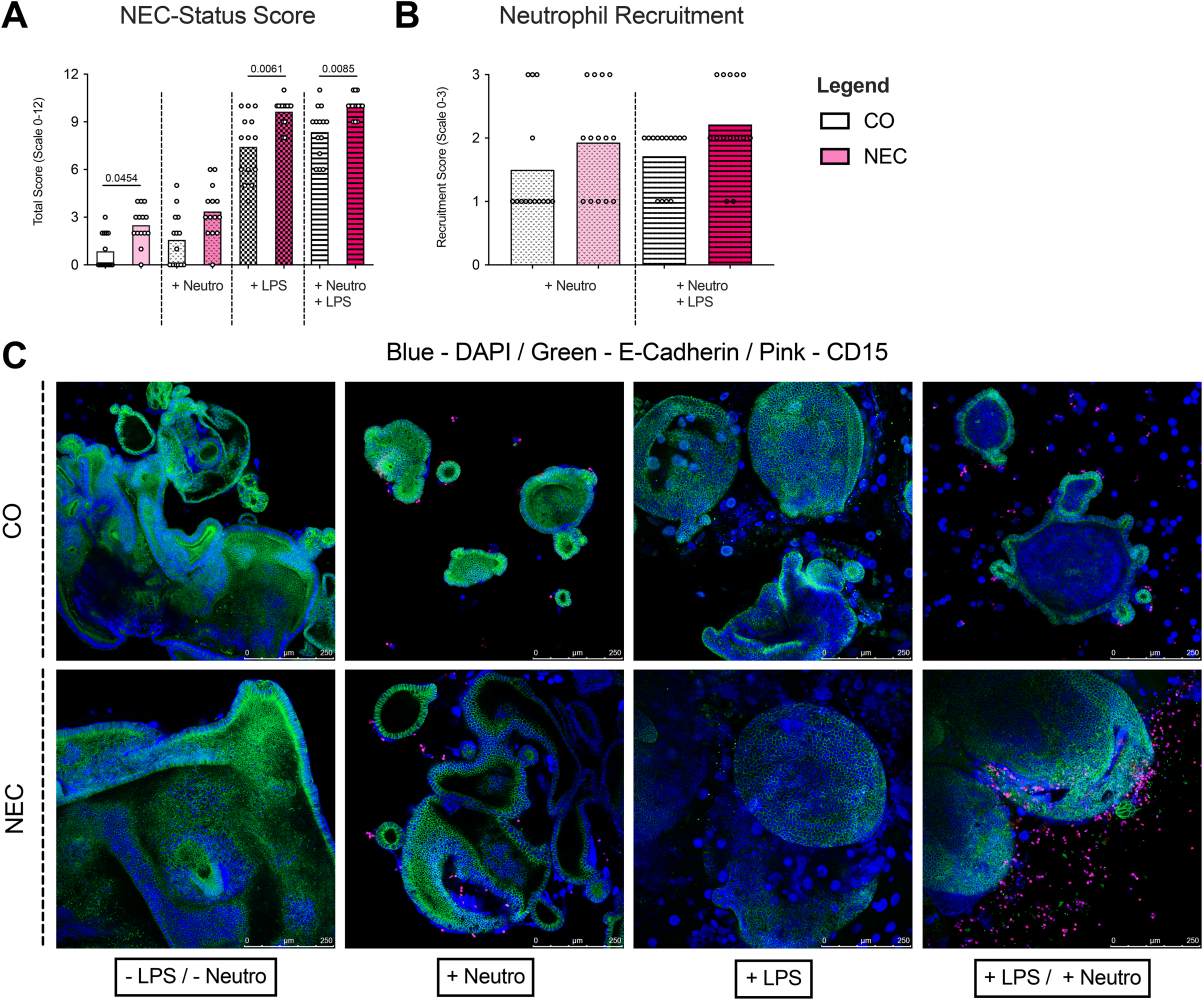
Immunofluorescence analyses and NEC-Score of neutrophil-organoid cocultures. **(A)** NEC scores were calculated based on immunofluorescence staining of neutrophil-organoid cocultures. A significant increase in NEC score was observed in cultures stimulated with LPS, indicating a stronger epithelial response to LPS-induced injury. **(B)** Neutrophil recruitment was assessed in cocultures with control and NEC organoids. A non-significant trend was observed toward increased neutrophil recruitment in NEC organoids upon LPS stimulation, suggesting that LPS may promote recruitment to NEC organoids but not to a significant extent compared to control organoids. **(C)** Representative immunofluorescence images of ileal tissue show the staining patterns of neutrophils and organoids in all experimental conditions. These images support the observed trends in recruitment and activation, with enhanced neutrophil presence in LPS-stimulated cocultures.

A semi-quantitative NEC-Status score was used to assess whether organoids derived from NEC tissue exhibited characteristics of NEC *in vitro*, revealing significantly higher scores (i.e. more disrupted cell borders, more broken nuclei, more cell debridement, and fewer mitoses) in untreated NEC organoids compared to CO organoids (2.50 ± 1.16 vs. 0.86 ± 1.10, p=0.0454). LPS stimulation increased the score in both NEC (9.64 ± 0.84) and CO (7.43 ± 2.07) organoids, with significantly higher values in NEC (p=0.0061). Neutrophil addition did not significantly alter NEC-Status scores in LPS-treated organoids or non-treated organoids (**Figure 4A**).

Immunofluorescence showed similar neutrophil recruitment to NEC and CO organoids under unstimulated conditions. LPS increased neutrophil recruitment in both organoid types, but no significant differences were seen between NEC and CO (**Figures 4B, C**).

## 4 Discussion

In this study, we generated a novel 3D tissue system to study NEC using neutrophil-intestinal organoid cocultures. Further, we described the characteristics of organoids derived from NEC and non-NEC tissues in coculture with neutrophils and upon stimulation with LPS. Epithelial cells of NEC organoids showed an increased rate of apoptosis and an elevated expression of the TLR-4 after stimulation with LPS. Specifically, significantly more cell damage, more cell debris, and fewer mitoses were observed in NEC-derived cultures compared to non-NEC tissues. In conclusion, the results suggest that epithelial cells from children with NEC have an elevated stress response upon coculture in organoids, particularly when additionally stressed with LPS.

Increased TLR-4 signaling has been indicated to importantly contribute to NEC, which is reflected in our model. Specifically, it is assumed that the neonatal intestinal mucosa of patients who develop NEC exhibits an impaired ability to accommodate the change from “sterility” to bacterial colonization (12, 19). To develop a physiologically healthy gut flora postpartum, it is essential that (1) an intestinal immune tolerance via proteolysis of the TLR-4 signaling molecules is achieved (22), and (2) epithelial cells are protected from bacterial impact (5). Both adaptations appear to fail in children with NEC. Further support for the pivotal role of TLR-4 in NEC is the fact that protective factors, such as breast milk, which suppress abnormal TLR-4 activation after birth, reduce the risk of NEC development (17, 22, 54). Using the *in vitro* neutrophil organoid system to model neutrophil epithelial cell interactions, we were able to show that NEC organoids had higher TLR-4 rates than healthy control organoids. However, it is not clear whether upregulated TLR-4 rates are the cause or result of NEC development. Supporting the role of TLR-4 upregulation as a causative agent for NEC is the hypothesis that children who go on to develop NEC might have an inherently elevated TLR-4 expression or dysregulation of TLR-4 postpartum. This hypothesis is supported by several genetic studies that have reported congenital genetic variants in the TLR-4 regulatory pathway contributing to the risk of NEC development (22). As an example, mutations in the SIGIRR gene, which is responsible for intestinal TLR-4 signaling and inhibition of inflammation induced by LPS, are reportedly partially associated with the development of NEC (21, 22, 55).

Once the NEC intestinal inflammation cascade, partially mediated by TLR-4 upregulation, manifests, neutrophils, as first-line responders of the innate immune system, begin to migrate to the site of inflammation (12, 23, 27). Thus, the role of neutrophils in NEC manifestation has been evaluated previously, with the result that neutrophils potentially play an important role in NEC (36–38). Using the *“in vitro* neutrophil organoid system” to model neutrophil epithelial cell interactions, this study suggests that even though neutrophils did not seem to trigger higher apoptosis rates when added to unstressed organoids (without LPS), irrespective of whether organoids were derived from CO or NEC affected tissue, an increase in intestinal organoid TLR-4 expression was observed when neutrophils were added to stressed organoids. Concurrently (1), neutrophils were more activated when cocultured with organoids regardless of NEC or control organoids, and (2) expressed higher TLR-4 levels, especially when added to NEC organoids, yet non-significant.

Based on the results, organoids and neutrophils interact, but neutrophils alone do not cause NEC manifestation. In contrast to their beneficial protective role in innate anti-microbial defense (56), neutrophils seem to aggravate the development of NEC. This conclusion is based on the higher recruitment rates of neutrophils in LPS-treated NEC cultures when compared to LPS-treated control cultures and higher TLR-4 expression in NEC cultures. As a result, LPS-treated NEC organoids with neutrophils displayed significantly higher NEC-Status scores in IF than control organoids. We propose that neutrophils have a more severe effect on intestines with high TLR-4 expression (as in NEC patients) than on intestines of healthy neonates and physiologically downregulated TLR-4.

Even though the findings of this study are promising, there are several limitations to consider. First, as intestinal organoids from organ-specific adult stem cells solely represent the epithelial cell layer (40), it is only possible to examine interactions among cells found within this layer and other independently isolated cells (in our case, neutrophils). While it is essential to investigate cell-to-cell interactions and the barrier system of the epithelial cells, other key components in NEC development, such as the immune system and various signaling pathways, can only be examined on an individual level. As such, results from *in vitro* cultures may not directly be translated to *in vivo* findings, as additional factors may play a role (57). Recently, this limitation was addressed by transplantation of organoids into mice (42, 58, 59). As a result, organoids can function in a complete *in vivo* system, including circulation and interaction with a plethora of immune cells. This is particularly important as the gut, with its microbiome, is an exceptionally unique and highly complex system (60, 61) with many features possibly contributing to NEC development (62–64). However, an *in vitro* 3D cell culture does provide the opportunity to adjust individual characteristics quickly and reproduce them more efficiently (65).

While a 3D organoid model provides a highly realistic, functional and versatile platform for structural studies including tissue differentiation, cell migration, and architectural changes under varying conditions (65–67), it may be beneficial to consider using a monolayer organoid model for examining direct luminal contact (66) with substances - like in our case LPS - or other cells of the immune system (44). In this scenario, the organoid monolayer would be oriented with its basolateral side facing down while the luminal side would be accessible for exposure, e.g. LPS or neutrophils (66).

Moreover, our study focused specifically on neutrophil granulocytes, given their essential role in the innate immune response and inflammation. However, to gain a more comprehensive understanding of NEC, it is important to also consider the involvement of other immune cells, including dendritic cells, B and T lymphocytes, macrophages, and others (28, 73, 74). In this context, future research could explore co-culture models that incorporate a broader range of immune cells to better investigate their interactions and collective impact on disease development.

Second, the samples examined in our study consisted of a mixture of colon- and ileum-derived cells. Both tissue types exhibit slightly different physiology and anatomy, which might also affect TLR-4 expression (68). Bearing this in mind, sub-analyses showed no significant differences between both intestinal entities. However, with bigger sample sizes, differences might become more apparent and should be considered in future research.

Third, based on our findings of the survival and activation of neutrophils in organoid differentiation medium and discoveries by Kolman et al., 2022, this study set the incubation period of organoids with neutrophils at 24 h (69). However, a longer incubation time might be necessary to mimic neutrophils’ effect on the intestinal organoids, as the development of fulminant NEC in humans can take longer than 24 hours (70, 71). This problem however could be overcome by adding more freshly isolated neutrophils after 24h to the coculture. With respect to neutrophil function, one must also consider possible co-factors necessary for neutrophils to uncover their full pathogenic potential (69), which should be included in future coculture models. Furthermore, as we were able to show in our comparison of neutrophil survival and activation levels in different media, neutrophils are highly vulnerable cells that quickly react to their surrounding media, possibly negatively affecting a coculture model when neutrophils’ needs are not fully met or the surrounding environment is not stable enough (72).

Fourth, it is essential to state that TLR-4 activation is enhanced by interferon-gamma, a proinflammatory factor produced by numerous immune cells (e.g., natural killer cells, T lymphocytes, and antigen-presenting cells) (75) and is hypothesized to sensitize TLR-4 to LPS (76). Consequently, the LPS response in our organoid model could potentially be improved and/or adapted to include new cellular and humoral players. This may exacerbate the NEC responses, making organoids even more applicable to studying human *in vivo* NEC pathophysiology.

Fifth, a further pathological feature of NETs, the occlusion of vessels (77, 78), especially those of the capillary field (79) is hard to investigate *ex vivo.* More sophisticated systems are required to analyze this aspect in *in vitro* models.

Last, one of the aims of this study was to develop a model that resembles human NEC as much as possible, in particular mimicking the tubular structure of the human bowel. As a result, we decided to experiment using NEC organoids in their sphere-like 3D formation. Nonetheless, using an organoid 3D formation could possibly pose a limitation to this study, as bacterial colonization of the gut normally occurs from the lumen of the bowel. In our organoid model, the LPS-containing media surrounded the organoids extraluminally. This limitation could likely be overcome by establishing a monolayer organoid culture (44), where both sides, apical and basolateral, can be reached by the LPS suspension (see point one in the limitation section). However, in doing so, the 3D structure would have to be dispensed, which is one of the key reasons to conduct intestinal organoid research.

In summary, our study established a 3D tissue system to model neutrophil-intestinal epithelial cell interactions in organoid cocultures. When exposed to LPS, NEC-derived organoids exhibited an exacerbated inflammatory response as measured via more NEC-like lesions, an elevated expression of TLR-4, and higher apoptosis rates compared to non-NEC organoids. These findings suggest a possible elevated stress response in children with NEC. Further, our results support the crucial role of TLR-4 in epithelial cells in NEC.

This novel neutrophil intestinal organoid system to model NEC provides an alternative to small animal models. In addition, the model could be used to test various treatment options for NEC, such as anti-NET therapies (37), while organoid cocultures with immune cells and medication might bridge the gap between animal and human research.

## Data Availability

The raw data supporting the conclusions of this article will be made available by the authors, without undue reservation.

## References

[1] Pierro A Hall N. Surgical treatments of infants with necrotizing enterocolitis. Semin Neonatol. (2003) 8:223–32. doi: 10.1016/S1084-2756(03)00025-3 15001141

[2] Pickard SS Feinstein JA Popat RA Huang L Dutta S. Short- and long-term outcomes of necrotizing enterocolitis in infants with congenital heart disease. Pediatrics. (2009) 123:e901–6. doi: 10.1542/peds.2008-3216 19403484

[3] Schnabl KL Van Aerde JE Thomson AB Clandinin MT. Necrotizing enterocolitis: a multifactorial disease with no cure. World J Gastroenterol. (2008) 14:2142–61. doi: 10.3748/wjg.14.2142 PMC2703838 18407587

[4] Neu J Walker WA. Necrotizing enterocolitis. N Engl J Med. (2011) 364:255–64. doi: 10.1056/NEJMra1005408 PMC3628622 21247316

[5] Anand RJ Leaphart CL Mollen KP Hackam DJ. The role of the intestinal barrier in the pathogenesis of necrotizing enterocolitis. Shock. (2007) 27:124–33. doi: 10.1097/01.shk.0000239774.02904.65 17224785

[6] Ryder RW Shelton JD Guinan ME. Necrotizing enterocolitis: a prospective multicenter investigation. Am J Epidemiol. (1980) 112:113–23. doi: 10.1093/oxfordjournals.aje.a112960 6772021

[7] Lin PW Stoll BJ. Necrotising enterocolitis. Lancet. (2006) 368:1271–83. doi: 10.1016/S0140-6736(06)69525-1 17027734

[8] Caplan MS. Neonatal necrotizing enterocolitis. Introduction. Semin Perinatol. (2008) 32:69. doi: 10.1053/j.semperi.2008.02.001 18346529

[9] Meister AL Doheny KK Travagli RA. Necrotizing enterocolitis: It’s not all in the gut. Exp Biol Med (Maywood). (2020) 245:85–95. doi: 10.1177/1535370219891971 31810384 PMC7016421

[10] Bazacliu C Neu J. Necrotizing enterocolitis: long term complications. Curr Pediatr Rev. (2019) 15:115–24. doi: 10.2174/1573396315666190312093119 30864508

[11] Hackam DJ Upperman JS Grishin A Ford HR. Disordered enterocyte signaling and intestinal barrier dysfunction in the pathogenesis of necrotizing enterocolitis. Semin Pediatr Surg. (2005) 14:49–57. doi: 10.1053/j.sempedsurg.2004.10.025 15770588

[12] Leaphart CL Cavallo J Gribar SC Cetin S Li J Branca MF. A critical role for TLR4 in the pathogenesis of necrotizing enterocolitis by modulating intestinal injury and repair. J Immunol. (2007) 179:4808–20. doi: 10.4049/jimmunol.179.7.4808 17878380

[13] Hackam DJ Sodhi CP Good M. New insights into necrotizing enterocolitis: From laboratory observation to personalized prevention and treatment. J Pediatr Surg. (2019) 54:398–404. doi: 10.1016/j.jpedsurg.2018.06.012 29980346 PMC6344311

[14] Blakely ML Lally KP McDonald S Brown RL Barnhart DC Ricketts RR. Postoperative outcomes of extremely low birth-weight infants with necrotizing enterocolitis or isolated intestinal perforation: a prospective cohort study by the NICHD Neonatal Research Network. Ann Surg. (2005) 241:984–9. doi: 10.1097/01.sla.0000164181.67862.7f PMC1359076 15912048

[15] Robinson JR Rellinger EJ Hatch LD Weitkamp JH Speck KE Danko M. Surgical necrotizing enterocolitis. Semin Perinatol. (2017) 41:70–9. doi: 10.1053/j.semperi.2016.09.020 PMC5777619 27836422

[16] Rich BS Dolgin SE. Necrotizing enterocolitis. Pediatr Rev. (2017) 38:552–9. doi: 10.1542/pir.2017-0002 29196510

[17] Nino DF Sodhi CP Hackam DJ. Necrotizing enterocolitis: new insights into pathogenesis and mechanisms. Nat Rev Gastroenterol Hepatol. (2016) 13:590–600. doi: 10.1038/nrgastro.2016.119 27534694 PMC5124124

[18] Martin CR Walker WA. Intestinal immune defences and the inflammatory response in necrotising enterocolitis. Semin Fetal Neonatal Med. (2006) 11:369–77. doi: 10.1016/j.siny.2006.03.002 16690363

[19] Nanthakumar NN Fusunyan RD Sanderson I Walker WA. Inflammation in the developing human intestine: A possible pathophysiologic contribution to necrotizing enterocolitis. Proc Natl Acad Sci U S A. (2000) 97:6043–8. doi: 10.1073/pnas.97.11.604 PMC18555 10823949

[20] Neal MD Sodhi CP Dyer M Craig BT Good M Jia H. A critical role for TLR4 induction of autophagy in the regulation of enterocyte migration and the pathogenesis of necrotizing enterocolitis. J Immunol. (2013) 190:3541–51. doi: 10.4049/jimmunol.1202264 PMC3608826 23455503

[21] Cuna A George L Sampath V. Genetic predisposition to necrotizing enterocolitis in premature infants: Current knowledge, challenges, and future directions. Semin Fetal Neonatal Med. (2018) 23:387–93. doi: 10.1016/j.siny.2018.08.006 PMC6626706 30292709

[22] Gomart A Vallee A Lecarpentier Y. Necrotizing enterocolitis: LPS/TLR4-induced crosstalk between canonical TGF-beta/wnt/beta-catenin pathways and PPARgamma. Front Pediatr. (2021) 9:713344. doi: 10.3389/fped.2021.713344 34712628 PMC8547806

[23] Hodzic Z Bolock AM Good M. The role of mucosal immunity in the pathogenesis of necrotizing enterocolitis. Front Pediatr. (2017) 5:40. doi: 10.3389/fped.2017.00040 28316967 PMC5334327

[24] Neal MD Leaphart C Levy R Prince J Billiar TR Watkins S. Enterocyte TLR4 mediates phagocytosis and translocation of bacteria across the intestinal barrier. J Immunol. (2006) 176:3070–9. doi: 10.4049/jimmunol.176.5.3070 16493066

[25] Sherman MP. New concepts of microbial translocation in the neonatal intestine: mechanisms and prevention. Clin Perinatol. (2010) 37:565–79. doi: 10.1016/j.clp.2010.05.006 PMC2933426 20813271

[26] Neal MD Jia H Eyer B Good M Guerriero CJ Sodhi CP. Discovery and validation of a new class of small molecule Toll-like receptor 4 (TLR4) inhibitors. PloS One. (2013) 8:e65779. doi: 10.1371/journal.pone.0065779 23776545 PMC3680486

[27] Hayashi F Means TK Luster AD. Toll-like receptors stimulate human neutrophil function. Blood. (2003) 102:2660–9. doi: 10.1182/blood-2003-04-1078 12829592

[28] Denning TL Bhatia AM Kane AF Patel RM Denning PW. Pathogenesis of NEC: Role of the innate and adaptive immune response. Semin Perinatol. (2017) 41:15–28. doi: 10.1053/j.semperi.2016.09.014 27940091 PMC5484641

[29] Selders GS Fetz AE Radic MZ Bowlin GL. An overview of the role of neutrophils in innate immunity, inflammation and host-biomaterial integration. Regener Biomater. (2017) 4:55–68. doi: 10.1093/rb/rbw041 PMC5274707 28149530

[30] Carvalho LO Aquino EN Neves AC Fontes W. The neutrophil nucleus and its role in neutrophilic function. J Cell Biochem. (2015) 116:1831–6. doi: 10.1002/jcb.v116.9 25727365

[31] Brinkmann V Reichard U Goosmann C Fauler B Uhlemann Y Weiss DS. Neutrophil extracellular traps kill bacteria. Science. (2004) 303:1532–5. doi: 10.1126/science.1092385 15001782

[32] Khandpur R Carmona-Rivera C Vivekanandan-Giri A Gizinski A Yalavarthi S Knight JS. NETs are a source of citrullinated autoantigens and stimulate inflammatory responses in rheumatoid arthritis. Sci Transl Med. (2013) 5:178ra40. doi: 10.1126/scitranslmed.3005580 PMC3727661 23536012

[33] Muller S Radic M. Oxidation and mitochondrial origin of NET DNA in the pathogenesis of lupus. Nat Med. (2016) 22:126–7. doi: 10.1038/nm.4044 26845404

[34] Weiss SJ. Tissue destruction by neutrophils. N Engl J Med. (1989) 320:365–76. doi: 10.1056/NEJM198902093200606 2536474

[35] Klinke M Chaaban H Boettcher M. The role of neutrophil extracellular traps in necrotizing enterocolitis. Front Pediatr. (2023) 11:1121193. doi: 10.3389/fped.2023.1121193 37009300 PMC10050739

[36] Vincent D Klinke M Eschenburg G Trochimiuk M Appl B Tiemann B. NEC is likely a NETs dependent process and markers of NETosis are predictive of NEC in mice and humans. Sci Rep. (2018) 8:12612. doi: 10.1038/s41598-018-31087-0 30135601 PMC6105661

[37] Klinke M Vincent D Trochimiuk M Appl B Tiemann B Bergholz R. Degradation of extracellular DNA significantly ameliorates necrotizing enterocolitis severity in mice. J Surg Res. (2019) 235:513–20. doi: 10.1016/j.jss.2018.10.041 30691836

[38] Klinke M Vincent D Trochimiuk M Appl B Tiemann B Reinshagen K. Development of an improved murine model of necrotizing enterocolitis shows the importance of neutrophils in NEC pathogenesis. Sci Rep. (2020) 10:8049. doi: 10.1038/s41598-020-65120-y 32415172 PMC7229036

[39] Woodcock J Woosley R. The FDA critical path initiative and its influence on new drug development. Annu Rev Med. (2008) 59:1–12. doi: 10.1146/annurev.med.59.090506.155819 18186700

[40] Rossi G Manfrin A Lutolf MP. Progress and potential in organoid research. Nat Rev Genet. (2018) 19:671–87. doi: 10.1038/s41576-018-0051-9 30228295

[41] Spence JR Mayhew CN Rankin SA Kuhar MF Vallance JE Tolle K. Directed differentiation of human pluripotent stem cells into intestinal tissue *in vitro* . Nature. (2011) 470:105–9. doi: 10.1038/nature09691 PMC3033971 21151107

[42] Chusilp S Li B Lee D Lee C Vejchapipat P Pierro A. Intestinal organoids in infants and children. Pediatr Surg Int. (2020) 36:1–10. doi: 10.1007/s00383-019-04581-3 31555860

[43] Kovler ML Sodhi CP Hackam DJ. Precision-based modeling approaches for necrotizing enterocolitis. Dis Model Mech. (2020) 13:3–8. doi: 10.1242/dmm.044388 PMC7328169 32764156

[44] Senger S Ingano L Freire R Anselmo A Zhu W Sadreyev R. Human fetal-derived enterospheres provide insights on intestinal development and a novel model to study necrotizing enterocolitis (NEC). Cell Mol Gastroenterol Hepatol. (2018) 5:549–68. doi: 10.1016/j.jcmgh.2018.01.014 PMC6009798 29930978

[45] Diaz L Zambrano E Flores ME Contreras M Crispin JC Aleman G. Ethical considerations in animal research: the principle of 3R’s. Rev Invest Clin. (2020) 73:199–209. doi: 10.24875/RIC.20000380 33090120

[46] Csukovich G Pratscher B Burgener IA. The world of organoids: gastrointestinal disease modelling in the age of 3R and one health with specific relevance to dogs and cats. Anim (Basel). (2022) 12:1–5. doi: 10.3390/ani12182461 PMC9495014 36139322

[47] Pillay J den Braber I Vrisekoop N Kwast LM de Boer RJ Borghans JA. *In vivo* labeling with 2H2O reveals a human neutrophil lifespan of 5. 4 days. Blood. (2010) 116:625–7. doi: 10.1182/blood-2010-01-259028 20410504

[48] Kulinska KI Szalkowska S Andrusiewicz M Kotwicka M Billert H. The effect of local anaesthetics on apoptosis and NETosis of human neutrophils *in vitro*: comparison between lidocaine and ropivacaine. Hum Cell. (2023) 36:2027–39. doi: 10.1007/s13577-023-00963-x PMC10587218 37589878

[49] Watt SM Sala-Newby G Hoang T Gilmore DJ Grunert F Nagel G. CD66 identifies a neutrophil-specific epitope within the hematopoietic system that is expressed by members of the carcinoembryonic antigen family of adhesion molecules. Blood. (1991) 78:63–74. doi: 10.1182/blood.V78.1.63.63 1712645

[50] Dransfield I Stocks SC Haslett C. Regulation of cell adhesion molecule expression and function associated with neutrophil apoptosis. Blood. (1995) 85:3264–73. doi: 10.1182/blood.V85.11.3264.bloodjournal85113264 7538822

[51] Alvarez-Larran A Toll T Rives S Estella J. Assessment of neutrophil activation in whole blood by flow cytometry. Clin Lab Haematol. (2005) 27:41–6. doi: 10.1111/j.1365-2257.2004.00661.x 15686506

[52] Ares GJ Buonpane C Yuan C Wood D Hunter CJ. A novel human epithelial enteroid model of necrotizing enterocolitis. J Vis Exp. (2019) 146. doi: 10.3791/59194-v PMC6814448 31033943

[53] Chiu CJ McArdle AH Brown R Scott HJ Gurd FN. Intestinal mucosal lesion in low-flow states. I. A morphological, hemodynamic, and metabolic reappraisal. Arch Surg. (1970) 101:478–83. doi: 10.1001/archsurg.1970.01340280030009 5457245

[54] Nolan LS Rimer JM Good M. The role of human milk oligosaccharides and probiotics on the neonatal microbiome and risk of necrotizing enterocolitis: A narrative review. Nutrients. (2020) 12:2–4. doi: 10.3390/nu12103052 PMC7600747 33036184

[55] Sampath V Menden H Helbling D Li K Gastonguay A Ramchandran R. SIGIRR genetic variants in premature infants with necrotizing enterocolitis. Pediatrics. (2015) 135:e1530–4. doi: 10.1542/peds.2014-3386 PMC4444800 25963006

[56] Liew PX Kubes P. The neutrophil’s role during health and disease. Physiol Rev. (2019) 99:1223–48. doi: 10.1152/physrev.00012.2018 30758246

[57] Saeidnia S Manayi A Abdollahi M. From *in vitro* Experiments to *in vivo* and Clinical Studies; Pros and Cons. Curr Drug Discov Technol. (2015) 12:218–24. doi: 10.2174/1570163813666160114093140 26778084

[58] Watson CL Mahe MM Múnera J Howell JC Sundaram N Poling HM. An *in vivo* model of human small intestine using pluripotent stem cells. Nat Med. (2014) 20:1310–4. doi: 10.1038/nm.3737 PMC4408376 25326803

[59] Watanabe S Kobayashi S Ogasawara N Okamoto R Nakamura T Watanabe M. Transplantation of intestinal organoids into a mouse model of colitis. Nat Protoc. (2022) 17:649–71. doi: 10.1038/s41596-021-00658-3 35110738

[60] de Vos WM Tilg H Van Hul M Cani PD. Gut microbiome and health: mechanistic insights. Gut. (2022) 71:1020–32. doi: 10.1136/gutjnl-2021-326789 PMC8995832 35105664

[61] Baranowski JR Claud EC. Necrotizing enterocolitis and the preterm infant microbiome. Adv Exp Med Biol. (2019) 1125:25–36. doi: 10.1007/5584_2018_313 30680646

[62] Neu J Pammi M. Pathogenesis of NEC: Impact of an altered intestinal microbiome. Semin Perinatol. (2017) 41:29–35. doi: 10.1053/j.semperi.2016.09.015 27986328

[63] Claud EC. Neonatal necrotizing enterocolitis -inflammation and intestinal immaturity. Antiinflamm Antiallergy Agents Med Chem. (2009) 8:248–59. doi: 10.2174/187152309789152020 PMC2874244 20498729

[64] Alganabi M Lee C Bindi E Li B Pierro A. Recent advances in understanding necrotizing enterocolitis. F1000Res. (2019) 8:3–5. doi: 10.12688/f1000research PMC6348433 30740215

[65] Kim J Koo BK Knoblich JA. Human organoids: model systems for human biology and medicine. Nat Rev Mol Cell Biol. (2020) 21:571–84. doi: 10.1038/s41580-020-0259-3 PMC7339799 32636524

[66] Criss ZK 2nd Bhasin N Di Rienzi SC Rajan A Deans-Fielder K Swaminathan G. Drivers of transcriptional variance in human intestinal epithelial organoids. Physiol Genomics. (2021) 53:486–508. doi: 10.1152/physiolgenomics.00061.2021 34612061 PMC8616596

[67] Silva-Pedrosa R Salgado AJ Ferreira PE. Revolutionizing disease modeling: the emergence of organoids in cellular systems. Cells. (2023) 12:4–7. doi: 10.3390/cells12060930 PMC10047824 36980271

[68] Price AE Shamardani K Lugo KA Deguine J Roberts AW Lee BL. A map of toll-like receptor expression in the intestinal epithelium reveals distinct spatial, cell type-specific, and temporal patterns. Immunity. (2018) 49:560–75 e6. doi: 10.1016/j.immuni.2018.07.016 30170812 PMC6152941

[69] Kolman JP Pagerols Raluy L Muller I Nikolaev VO Trochimiuk M Appl B. NET release of long-term surviving neutrophils. Front Immunol. (2022) 13:815412. doi: 10.3389/fimmu.2022.815412 35242132 PMC8887621

[70] Gephart SM McGrath JM Effken JA Halpern MD. Necrotizing enterocolitis risk: state of the science. Adv Neonatal Care. (2012) 12:77–87. doi: 10.1097/ANC.0b013e31824cee94 22469959 PMC3357630

[71] Schurink M Kooi EM Hulzebos CV Kox RG Groen H Heineman E. Intestinal fatty acid-binding protein as a diagnostic marker for complicated and uncomplicated necrotizing enterocolitis: a prospective cohort study. PloS One. (2015) 10:e0121336. doi: 10.1371/journal.pone.0121336 25793701 PMC4368100

[72] Gonzalez Gonzalez M Cichon I Scislowska-Czarnecka A Kolaczkowska E. Challenges in 3D culturing of neutrophils: Assessment of cell viability. J Immunol Methods. (2018) 457:73–7. doi: 10.1016/j.jim.2018.02.015 29476762

[73] Peterslund P Rasmussen L Qvist N Hansen TP Husby S Detlefsen S. Frequencies of immune cells in the human small bowel during normal gestation and in necrotizing enterocolitis. Fetal Pediatr Pathol. (2019) 38:153–66. doi: 10.1080/15513815.2018.1561774 30689475

[74] Liu J Joseph S Manohar K Lee J Brokaw JP Shelley WC. Role of innate T cells in necrotizing enterocolitis. Front Immunol. (2024) 15:1357483. doi: 10.3389/fimmu.2024.1357483 38390341 PMC10881895

[75] Schroder K Hertzog PJ Ravasi T Hume DA. Interferon-gamma: an overview of signals, mechanisms and functions. J Leukoc Biol. (2004) 75:163–89. doi: 10.1189/jlb.0603252 14525967

[76] Suzuki M Hisamatsu T Podolsky DK. Gamma interferon augments the intracellular pathway for lipopolysaccharide (LPS) recognition in human intestinal epithelial cells through coordinated up-regulation of LPS uptake and expression of the intracellular Toll-like receptor 4-MD-2 complex. Infect Immun. (2003) 71:3503–11. doi: 10.1128/IAI.71.6.3503-3511.2003 PMC155722 12761135

[77] Yaykasli KO Schauer C Munoz LE Mahajan A Knopf J Schett G. Neutrophil extracellular trap-driven occlusive diseases. Cells. (2021) 10:1–4 . doi: 10.3390/cells10092208 PMC8466545 34571857

[78] Singh J Boettcher M Dolling M Heuer A Hohberger B Leppkes M. Moonlighting chromatin: when DNA escapes nuclear control. Cell Death Differ. (2023) 30:861–75. doi: 10.1038/s41418-023-01124-1 PMC9907214 36755071

[79] Singh J Herrmann I Mahajan A Schauer C Shan X Hartmann A. A pleomorphic puzzle: heterogeneous pulmonary vascular occlusions in patients with COVID-19. Int J Mol Sci. (2022) 23. doi: 10.3390/ijms232315126 PMC9739020 36499449

